# A mHealth Application for Chronic Wound Care: Findings of a User Trial

**DOI:** 10.3390/ijerph10116199

**Published:** 2013-11-19

**Authors:** Marcia R. Friesen, Carole Hamel, Robert D. McLeod

**Affiliations:** 1 Design Engineering, University of Manitoba, E2-262 EITC, Winnipeg, Manitoba R3T 5V6, Canada; 2 Riverview Health Centre, 1 Morley Ave., Winnipeg, Manitoba R3L 2P4, Canada; E-Mail: chamel@rhc.mb.ca; 3 Electrical and Computer Engineering, University of Manitoba, E2-390 EITC, Winnipeg, Manitoba R3T 5V6, Canada; E-Mail: Robert.McLeod@UManitoba.ca

**Keywords:** pressure ulcers, chronic wounds, eHealth, mHealth, telehealth

## Abstract

This paper reports on the findings of a user trial of a mHealth application for pressure ulcer (bedsore) documentation. Pressure ulcers are a leading iatrogenic cause of death in developed countries and significantly impact quality of life for those affected. Pressure ulcers will be an increasing public health concern as the population ages. Electronic information systems are being explored to improve consistency and accuracy of documentation, improve patient and caregiver experience and ultimately improve patient outcomes. A software application was developed for Android Smartphones and tablets and was trialed in a personal care home in Western Canada. The software application provides an electronic medical record for chronic wounds, replacing nurses’ paper-based charting and is positioned for integration with facility’s larger eHealth framework. The mHealth application offers three intended benefits over paper-based charting of chronic wounds, including: (1) the capacity for remote consultation (telehealth between facilities, practitioners, and/or remote communities), (2) data organization and analysis, including built-in alerts, automatically-generated text-based and graph-based wound histories including wound images, and (3) tutorial support for non-specialized caregivers. The user trial yielded insights regarding the software application’s design and functionality in the clinical setting, and highlighted the key role of wound photographs in enhancing patient and caregiver experiences, enhancing communication between multiple healthcare professionals, and leveraging the software’s telehealth capacities.

## 1. Introduction

Pressure ulcers, also known as bedsores or decubitus ulcers, are a common but preventable condition seen most often in elderly persons and people with limited mobility. Pressure ulcer incidence rates vary widely from facility to facility, but the Canadian Association of Wound Care reports that one in four people in any healthcare facility have a pressure ulcer at any given time (25% in acute care, 30% in non-acute care, 22% in mixed health-care settings, and 15% in community care) [1,2]. 

Pressure ulcers are a disabling condition with numerous impacts on patient health and quality of life. Pressure ulcers are often a secondary condition that develops upon admission to hospital, and can lengthen hospital stay, delay the recovery of the patient, and exacerbate mobility limitations and social isolation as part of overall negative social and psychological impacts [3]. Pressure ulcers are also associated with increased mortality rates from underlying conditions [4,5,6,7,8], and the financial impact of treating pressure ulcers is substantial [9]. 

Common interventions to prevent and treat pressure ulcers include appropriate support surfaces, regular repositioning of the patient, optimizing nutrition, and skin care [10]. Additionally, risk assessment and regular standardized documentation are identified as critical steps in the prevention and treatment of pressure ulcers [11], although challenges are non-compliance to protocol and inconsistency of documentation. Data are often lacking and existing information is not specific enough [12] or lacks completeness [13], in part due to standardized forms that are overly lengthy and cumbersome for healthcare workers already taxed for time. 

Increasingly, attention is focused on electronic information systems as one part of an overall strategy of pressure ulcer prevention and treatment. This is a natural outgrowth of eHealth and mHealth initiatives whose intended outcomes are promoting patient safety, improving patient outcomes, enhancing financial and other efficiencies, and facilitating communication between multiple partners [9]. More specifically, electronic medical records (EMR) have shown promise in increasing quality of care, decreasing utilization of care, but have demonstrated mixed results relative to cost impacts [14]. In one study, an EMR system specific to chronic wound treatment has shown promise to simplify the evaluation and treatment of chronic wounds, although a standard protocol for wound images was vital to the value of the system [9]. Another study evaluated the costs and potential savings associated with home telehealth in the treatment or prevention of pressure ulcers, finding that cost savings were realized when low-cost technologies were installed (*vs*. high-cost and interactive technologies) and that home telehealth could reduce the prevalence of stage III and IV pressure ulcers [15]. In other existing wound EMR systems, problems that hindered their effective implementation included redundancy, lack of thoroughness, lack of flexibility, absence of standardized vocabulary, and non-mobility of the EMR [16]. 

While electronic documentation is certainly not the sole or defining factor between poor patient outcomes and good patient outcomes, electronic documentation can potentially contribute to more effective communication and collection of patient information, resulting in more effective patient care [17]. This research follows this conjecture that better compliance in documenting wound care, higher consistency in how a wound is documented, and higher frequency in wound monitoring are only a few factors that contribute to better health outcomes. 

A broad review demonstrates numerous eHealth technologies being developed for the healthcare community, although they are not well-catalogued. Relative to wound care, MediSense offers wound care software on a web-based interface on a computer monitor [18]. A product most similar to the current work is WoundRounds, developed in the United States [19]. Similar to our work, WoundRounds is a software application running on a Smartphone or handheld device. In summer 2013, WoundMAP Pump, Ulcercare, and Wound Mender entered as newcomers to the range of wound care apps, in various stages of development [20]. Our work offers the benefit of tailoring the application to the facility-specific wound care forms and other facility-specific preferences and IT infrastructure. 

Previous work in our research group has resulted in the development of a prototype mHealth application (software application for mobile devices) by which nurses and other healthcare providers can document patients’ chronic wounds and their treatments [21]. The wound care app was developed for Android Smartphones and tablet devices, and is intended for eventual use by healthcare providers in hospital and personal care homes and by home-based caregivers (homecare provider or family member) caring for a patient within the residence. This paper reports on the findings of a user trial of the prototype application with nurses who voluntarily participated in using the wound care app with patients in a healthcare facility in Winnipeg, Canada. The objective of the user trial was to receive feedback on the design and functionality of the app and to assess caregivers’ experiences in using the wound care app. While the primary objective of this paper is to report findings of the user trial, the prototype software application is presented first in order to establish the necessary context within which to understand the user trial findings. 

## 2. System Design

The wound care app was designed to replicate the paper-based charting currently used in the Winnipeg Regional Health Authority (WRHA), a publicly-funded system of healthcare services and facilities. The WRHA provides health care to more than 700,000 people within the region, as well as support and specialty referral services to nearly 500,000 people who live outside the region. The WRHA operates or funds over 200 health service facilities including hospitals, personal care homes, and home care services, which in turn employ approximately 28,000 people working within the health region.

The wound care app allows nurses to enter a new patient record, view an existing patient record, enter and assess new wounds on patients, and re-assess existing wounds on patients using the Pressure Ulcer Scale for Healing (PUSH tool) [22], and Braden Scale [23] and the Bates-Jensen tool [24]. Each Smartphone or tablet device running wound care software is associated with an individual healthcare worker and their multiple patients (rather than the device being associated with a unique patient at the bedside and their multiple healthcare providers). General objectives were to design an interface that would maximize user compliance and the value of the data for primary users. This included simplicity of the user interface, minimizing visual elements on any given screen to reduce clutter, using color cues to focus information, and converging on critical information. The design minimizes the number of steps required to complete common tasks (wound entry, wound assessment), and intuitive guidance leads the user only to the areas of the form applicable for the given patient. The application is designed with minimal opportunities for free-lance comments in light of the small screen size and objectives to standardize data entry and reduce input errors. Simple widgets (checkboxes and spinners) are used for data entry whenever possible. Additional functionality considerations include the potential discomfort or inconvenience of carrying the device on one’s person, maintaining its battery capacity, considerations in infection control, and the role of the data as part of the legal medical record. 

In addition to replicating the paper-based charting used in the WRHA, the app was designed for three intended benefits relative to paper-based charting of wounds and wound care:

(1) *Telehealth*: The potential to share data over cellular and Wi-Fi networks within and across facilities, for expert consultation and data sharing among multiple caregivers. Use cases include caregivers in remote communities seeking consultation and support from wound care specialists in major centres, and caregivers within a major centre reducing the need to transport patients for specialized consultation. In both cases, considerable patient physical and emotional stress, time, and expense can be avoided by keeping the patient in their long-term environment and sharing data via the app’s teleheath capacities. In Canada, where the population is dispersed over a very wide geographical area, telehealth is a significant benefit for those living in the rural and Northern communities, which are often serviced by a nursing station or health clinic with only limited staff and equipment resources.
(2) *Data Organization and Interpretation*
  a. Alerts: The app generates alerts for the caregiver upon login, notifying the caregiver to wounds that are in need of re-assessment and wounds that have been deteriorating from one assessment to the next. The user can individualize the alert criteria for time between assessments and the criteria for deterioration within the app
  b. Wound histories in graphical and text-based formats, including wound images: The app automatically generates a history of key wound indicators for all previous assessment, displaying this history in both a graph format and a text format. Where devices have built-in, back-facing cameras, wound images can be incorporated into the wound histories
(3) *User support* through help screens with definitions of specialized terms, tutorials, and simulated patients. This feature acknowledges that wound care is a specialized area of nursing and that not all caregivers have the specialized training to accompany their role.

**Figure 1:**
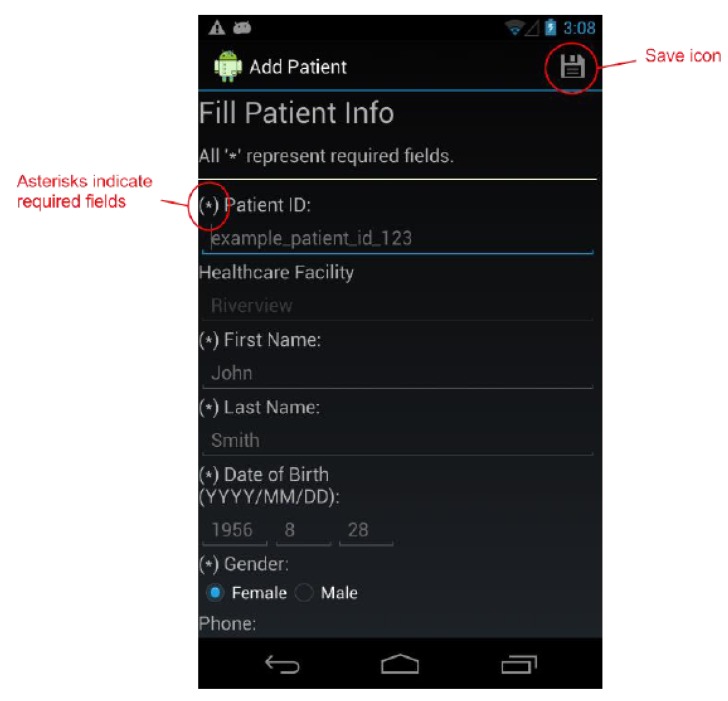
The *Add Patient* Screen.

**Figure 2:**
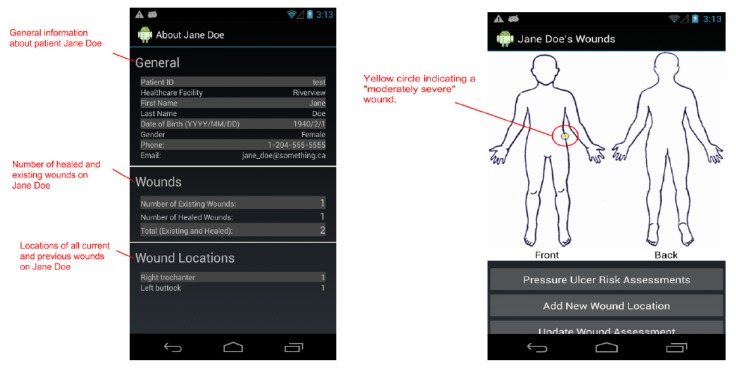
The *Patient Info* Screen.

**Figure 3:**
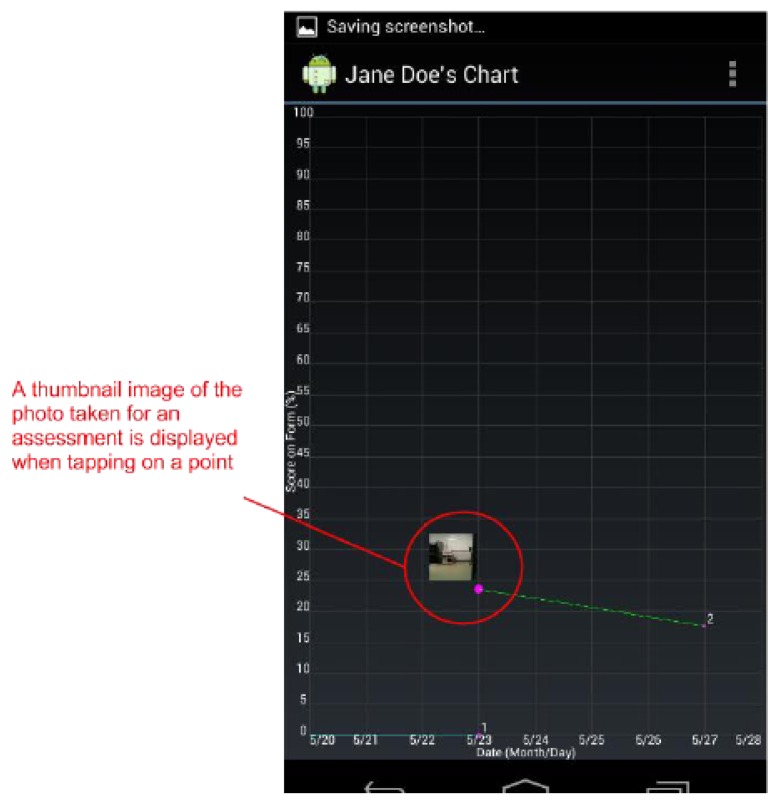
The *Wound Graph* screen plots all wound assessment scores on a graph. Tapping on line vertices (points) will display a thumbnail image of the photo taken for that assessment.

**Figure 4:**
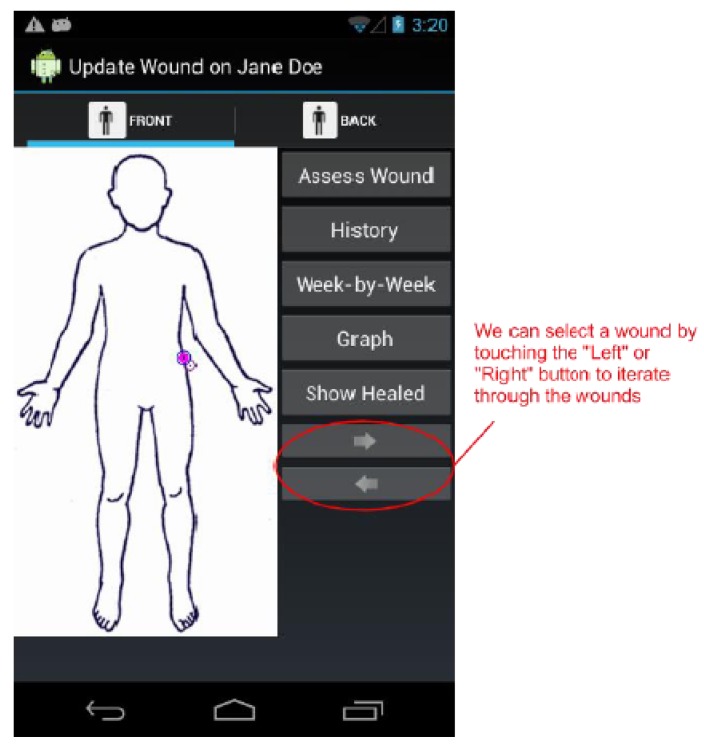
The *Update Wound Assessment* screen. We can select wounds by either using the cursor on the patient image, or by tapping the “Left” and “Right” buttons. Once a wound is selected, we may reassess the wound, view its history, compare the last two assessment forms, or view graphically view all of its assessment scores.

**Figure 5:**
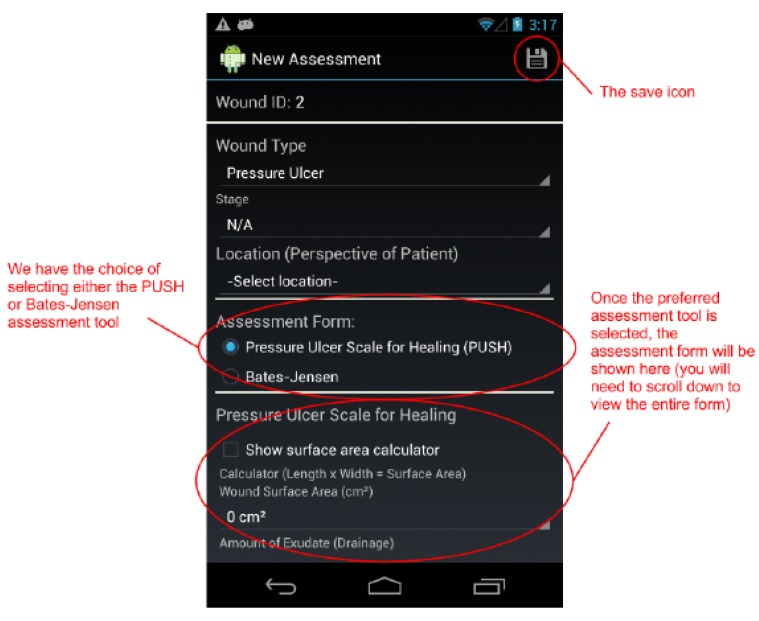
The top of the *New Assessment* screen. We have the choice of selecting either the PUSH or Bates-Jensen assessment tool to assess a pressure ulcer.

**Figure 6:**
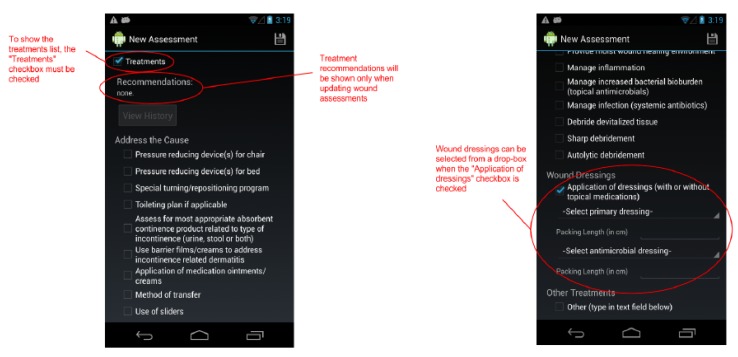
The *New Assessment* screen continued. A large list of treatments may be selected when the “Treatments” checkbox is checked. Recommendations may be provided when reassessing a wound.

**Figure 7:**
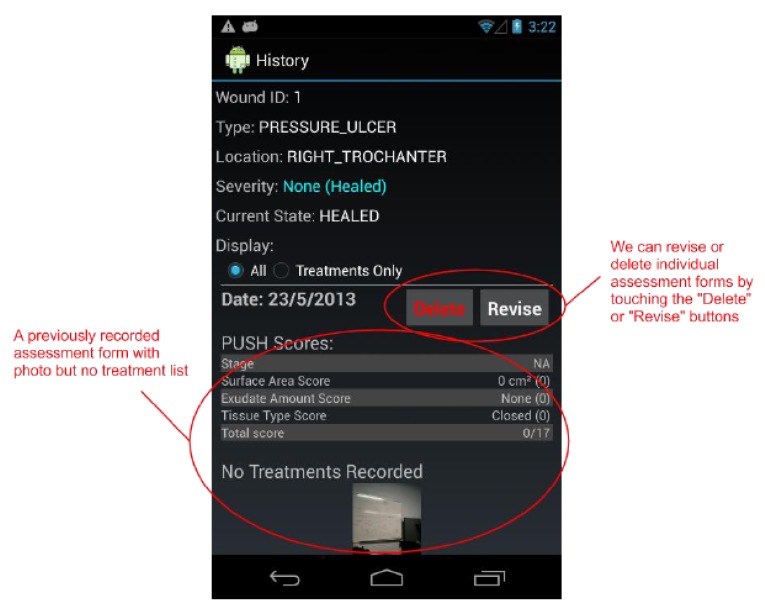
The *Wound History* screen shows us every assessment recorded (including treatments list and photo) for a selected wound. We may also *delete* and *revise* individual assessments from this screen.

**Figure 8:**
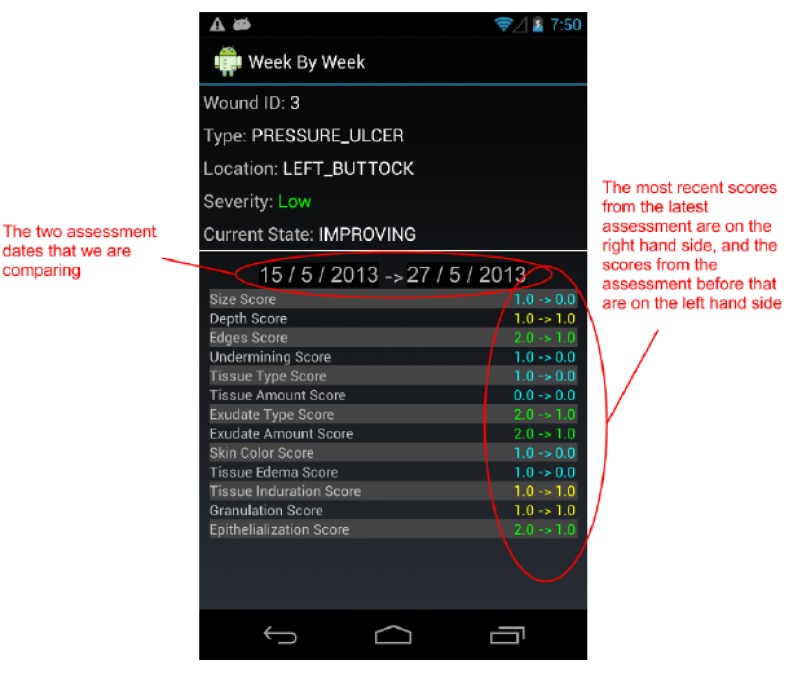
The *Week-by-Week Comparison* screen compares the last two assessments for a selected wound in more detail. It compares the score for every field in the two assessment forms.

By design, a benefit of the wound care software app is its potential as a stand-alone electronic medical record (EMR) and its positioning for integration into a facility-wide or region-wide EMR system. Privacy of personal health information and medical records is a significant priority. Each user is assigned a user ID and password for a secure login. In a full-scale implementation, access rights are confirmed via a 3G/4G or Wi-Fi secure connection to a server. The server will hold all patients’ wound data and users’ information. All user IDs and passwords will only be granted from an administrative standpoint, run by a separate server-side application. For added security, all messages sent from either client or server will be encrypted. If remote server infrastructure is not available or not desirable, the software application can be used with the Smartphone or Tablet’s internal memory card to keep a patient’s record private to the device itself. Having one centralized server (whether on-site, off-site, and/or shared between multiple facilities) allows for privileged, server-side applications to mine the data for anomalies within and between data sets. Being IP centric, all public internet security protocols would be integrated. Representative screenshots of the wound care app are shown in Figure 1, Figure 2, Figure 3, Figure 4, Figure 5, Figure 6, Figure 7 and Figure 8. 

## 3. User Trial

Upon approval of the University’s research ethics board and the healthcare facility’s research board, a user trial was implemented in 2013 at Riverview Health Centre (RHC) in Winnipeg, Canada. Riverview Health Centre is a 387-bed facility that caters to adults with rehabilitation, palliative and long term care needs in hospital and personal care home units, as well as outpatient and community programs. The Centre provides specialized services which include geriatric rehabilitation, palliative care, brain injury and stroke rehabilitation, behaviour management, long-term care, and complex continuing care.

A general invitation to participate in the user trial was sent to all nursing staff at RHC, and interested participants were invited to contact a member of the research team for further information. All participation was voluntary and was not compensated. After preliminary information sessions, eight nurses remained interested in participating in the trial. 

The nurses were all employees of Riverview Health Centre who regularly cared for patients with pressure ulcers and other chronic wounds. Four participants had up to 10 years’ nursing experience and the remainder had over 15 years’ nursing experience in personal care homes. Approximately half of participants assessed themselves to be “very tech-savvy”, while the other half declared themselves to be “comfortable with common features of phones and tablets”. Participants’ comfort with Smartphone / tablet interfaces and with touchscreens was self-assessed at 4.57/5.00 (range = 4.0–5.0; SD = 0.53) and 4.71/5.00 (range = 4.0–5.0; SD = 0.49), respectively. Three participants were male and five participants were female. The ages of participants ranged from 31–60, with respondents reporting their ages by decade (two respondents aged 31–40; three respondents aged 41–50; three respondents aged 51–60). All respondents reported working five or more shifts per week, and all respondents reported that each shift was five to eight hours in duration. To preserve anonymity of participants, these aggregate characteristic are purposely not cross-referenced to one another. 

These nurses were invited to a training session in which they were provided with either a new Nexus 4 Smartphone (four nurses) or a new Nexus 7 tablet (four nurses) with the wound care app loaded, a training manual for the wound care app, and were walked through a 90-minute training and demonstration of the app by the app developer and other members of the research team. After this training session, the nurses took the devices home and learned the app further on their own. They were invited to contact any member of the research team at any time for technical questions or other questions or concerns about the study.

Approximately 10 days after the training session, the nurses were asked whether another training session with all or some of them was desired, and no need for additional training was expressed nor identified. While this alone does not confirm comfort and fluency with the software application, the nurses’ preparation to use the app was later confirmed in the follow-up survey, the focus group, and in the nature of the questions that were raised to the developer during the user trial itself. The questions raised with the developer related to minor functions of the app which would require a good working knowledge of the overall framework. 

The nurses then began to use the app in their day-to-day nursing practice, with patients who had wounds and who had provided consent either themselves or through a designate. Nurses were asked to use the app for a minimum of seven consecutive nursing shifts, and were encouraged to use the app for a longer period of time if possible. Due to varying nursing schedules, the effects of part-time *vs.* full-time work, and summer vacation schedules, one participant had finished collecting data in 10 days, and the longest period of time used by a participant to collect data was two-and-a-half months. 

The use represented an additional workload over and above the participants’ regular nursing duties. Because the app is still in development, it did not replace but rather it replicated the paper-based charting that forms the official medical record of the patient. In using the app, the nurses were duplicating their paper-based charting for documentation of chronic wounds and care of wounds with the software on the Nexus Smartphone or tablet. The findings are acknowledged to reflect the varied experiences of nurses who voluntarily participated in the user trial and who each used the app in their own unique way, just as they would each be charting their patients on paper with their own unique tendencies, habits, and preferences of order. Thus, the study was designed to represent a very authentic use of the app over time in a naturalistic setting. While a quantitative research paradigm may perceive a lack of control in the study, the design complies with qualitative research norms, in which data and interpretations of data are validated by using triangulation and member checks. 

Once the nurses had been using the app for approximately three weeks, the primary researcher made an on-line survey accessible to the nurses (Supplementary file). The nurses were asked to complete an anonymous on-line survey at the time that they had completed the minimum (or more) nursing shifts with the app, in order to gather their immediate impressions of the app’s design and functionality. The on-line survey, developed in SurveyMonkey^®^, asked for the nurses’ impressions of the app’s features, content, navigation, and intended benefits. The survey used both open-ended and close-ended responses and was designed to explore the functionality and ease of use of the app, the integrity of the app’s content relative to paper-based forms, and the caregiver’s experience in using the app for wound care documentation. The survey remained available until all nurses had a chance to complete it. The primary researcher then summarized the survey results and provided a summary back to the participants for review and comment. 

Approximately six weeks after the nurses had finished using the app, a focus group session was held with the nurses and the research team. The focus group, approximately 75 minutes long, was used to probe into the survey results more deeply. Because the variable nursing schedules led to some nurses completing their minimum number of shifts weeks earlier than other nurses, the focus group followed directly on the day-to-day use for some of the eight nurses and with several weeks of elapsed time since day-to-day use for others of the eight nurses. This difference is recognized and is not considered to compromise the findings of the focus group. Rather, the findings encompass both the immediate and the long-term impressions of the app’s features and intended benefits, both of which are valuable to assess functionality. 

The focus group findings were likewise summarized and were provided back to the participants for review and comment. Subsequently, the researchers used the survey findings and focus group findings to delineate the key design issues that needed to be addressed in the app in further development. 

## 4. Findings

The objectives of the survey and the focus group were to obtain feedback on the design and functionality of the app and to investigate the nurses’ experiences in using the app. The user trial was not designed to directly investigate the patients’ experiences with the app nor was it designed to investigate patients’ clinical outcomes related to wounds. To ensure compliance with research ethics guidelines for data privacy, data was stored on the device itself rather than in a central server with remote access, as would be envisioned in a full-scale implementation of the app in a healthcare facility. The main numerical findings discussed in this section are summarized in Table 1.

**Table 1:** Main Numerical Survey Findings.

Survey Parameter All parameters are ranked on a likert-type scale from 1.0 (low) to 5.0 (high)	Mean score	Range	Standard deviation
How well-matched is the scope and depth of the software application to the Braden Scale tool?	4.60	4.0–5.0	0.55
How well-matched is the scope and depth of the software application to the PUSH tool?	4.57	4.0–5.0	0.53
Ease of entering a new patient record	4.57	4.0–5.0	0.53
Ease of finding my existing patient’s/resident’s wound record	4.71	4.0–5.0	0.49
Ease of adding a new wound to the patient’s record	4.50	3.0–5.0	0.84
Ease of assessing a new wound for the first time	4.57	3.0–5.0	0.79
Ease of assessing an existing wound that had been previously assessed	4.29	2.0–5.0	1.11
Screens were presented in an expected and logical order	4.17	3.0–5.0	0.75
Text History: This presentation is easy to understand.	4.50	4.0–5.0	0.55
Text History: This presentation is helpful in understanding wound progression.	4.50	4.0–5.0	0.55
Text History: This presentation adds to my understanding of the history of the patient’s/resident’s wounds and wound care, compared to not having this text-based history available.	4.50	4.0–5.0	0.55
Graph History: This presentation is easy to understand.	3.67	2.0–5.0	1.03
Graph History: This presentation is helpful in understanding wound progression.	3.83	3.0–5.0	0.75
Graph History: This presentation adds to my understanding of the history of the patient’s/resident’s wounds and wound care, compared to not having this graph-based history available.	3.67	2.0–5.0	1.03

In general, the participants reported that the wound care app was straight-forward to learn, well-matched to paper forms for wound care, and easily integrated into day-to-day nursing practices. Participants who used Smartphones (Nexus 4) during the user trial reported that they were easy to carry but that text size suffered; tablet users reported that text size and readability was not a problem, but that the device was bulkier to carry in the pockets of their nursing uniforms. Participants reported a high degree of ease in how the app guided users to enter a new patient record (4.57/5.00), find an existing patient record (4.71/5.00), add a new wound to an existing patient record (4.50/5.00), assess a wound for the first time (4.57/5.00), and assess an existing wound that had been previously assessed (4.29/5.00). Participants reported that the wound care app presented screens to them in an expected and logical order (4.17/5.00).

Based on survey results and focus group responses, it was evident that certain software functionalities have become standard expectations by users, regardless of platform or customization. In particular, the participants identified the need for more cross-navigation between various areas of the app, more standard ways to confirm deletion of an entry and undo an accidental deletion of an entry, and auto-saving of entries when navigating away from a given screen. Although these features all existed in the custom-designed app, their implementation—to the extent that it was not the same as standard software (e.g., Microsoft interfaces)—was at times found to be confusing. A number of other minor design and functionality improvements were recommended and future development will address these features. 

Participants reported a very strong correlation between the paper-based forms and the wound care app in terms of content and data entry expectations, with scores of 4.60/5.00 for the Braden Scale and 4.57/5.00 for the PUSH tool. The app was designed to provide intuitive guidance, only displaying the required data fields based on the nurse’s prior data entry. Participants reported that the intuitive guidance accurately reflected the fields necessary for a given patient and their wound condition. 

Participants reported that they did not use the Treatments section of the software application, and therefore, no meaningful data on the Treatments section of the software application was obtained. The Treatments section was designed to include all the possible treatments listed on the paper forms, appropriately categorized and sectioned. Nevertheless, the participants indicated that on a small Smartphone or tablet screen, the list was perceived as being too lengthy and included too many items to scroll through in order to find the three to five standard treatments regularly used with patients. This finding points to a design issue that will be addressed in subsequent versions of the software. 

The survey and the focus group also explored participants’ experiences and impressions of the intended benefits of the wound care app. The wound histories were identified as a positive feature for both the nurses and physicians who were consulted on a wound, although their value and use was tempered by the relatively short timeframe of the user trial. Participants found text-based histories more valuable (4.50/5.00) than graph-based histories (3.72/5.00) where data points were reportedly difficult to discern (a design issue that can be easily rectified). While an interesting result, this difference (4.50 *vs.* 3.72) did not prove to be statistically significant (*p* = 0.05). Furthermore, participants were unanimous in their view that the wound histories would have been even more valuable in a longer user trial. Although all participants in the user trial were experienced wound care nurses, the findings also demonstrated a strong desire for even more tutorial support than the app currently offered. A comprehensive listing of definitions of specialized terms was identified as a requirement in the app, although the scope of what constituted a “specialized term” at times including terms that the research team had considered to be a part of general nursing practice. 

The alert features were met with mixed perceptions. Currently, the alerts are displayed upon login in the Main Menu page. They are noted as listed items (New Assessments Needed (x) and Deteriorating Wounds (x), where x represents the number of patients to which the alert applies) among other listed items that include options to Add New Patient, Load Patient Data (for existing patient records), View All Patients, view Your Profile, and Logout. Some participants reported that they would regularly overlook the alert lines because their attention was focussed on the other options on the main menu. A design modification will be to highlight the alerts through a combination of unique font, text color, and size, and visual placement in an SMS-message type format upon login. 

Wound images (photographs) strongly emerged as the key value and benefit of the wound care app as experienced during the user trial. Participants’ feedback on the survey and in the focus group revealed benefits for the caregiver, the patient and their family, and for the physician and allied health professionals. Participants indicated that they appreciated and enjoyed taking pictures of the wound, and showing the photograph to the patient. Participants noted that being able to show a wound photograph to the patient was particularly useful when the wound was in a location that the patient would otherwise not be able to see, such as the buttocks, back of the legs or under the foot. Several participants noted that patients and their families appreciated seeing the wound and in doing so, gained a new appreciation of the instructions they had been given regarding hygiene and dressings in caring for their wound. Participants reported that in day-to-day practice, it can take up to 20 min to treat and re-dress a wound, and then a physician or allied health professional may enter the room and ask to view the wound. In the user trial, participants reported that physicians found the wound image sufficient for their purposes and did not require the wound to be undressed for visual inspection. Further, participants reported that the wound image was useful in augmenting a verbal description of the wound in a group consult away from the patient’s room. By using wound images with physicians, wound care consultants, and other consulting partners, the number of dressing changes of a wound can be reduced, and this in turn promotes wound healing. These findings supported the value of both the wound history and the telehealth features of the wound care app. 

Participants noted that the value of the wound image relies on consistent implementation of protocols for taking photographs (lighting, distance from wound, *etc.*), and the researchers acknowledge that more work is required to assess the extent to which a wound can be accurately assessed via digital images. Nurses in this user trial noted that devices that only have a front-facing camera (a camera that faces the user of the device) will present much more difficulty in setting up a wound photograph. Wound photography has already been recognized for its value in the care of other types of chronic wounds [25,26], including the evaluation of pressure ulcers via videoconferencing in which the observations reasonably approximated those obtained via an in-person assessment [27]. The findings in the present work resonate with other findings on wound photography, which highlight the importance of parameters that include, but are not limited to, equipment, photomacrography, camera lens orientation relative to the wound, flash settings to ensure consistent lighting, and duplicate photographs [9]. Ongoing work is focussed on the development of image analysis algorithms for detection and analysis of wound size and chromaticity, in order to correlate these to other wound information and add to the intelligence and range of information that the app can offer users. 

## 5. Conclusion

The wound app framework was designed to facilitate extensions to other platforms (iOS, BlackBerry, and device-agnostic HTML5 framework) and to other wounds (e.g., surgical wounds). Additional extensions can include the reporting of other prevalent community health issues, including monitoring of blood pressure, body weight, blood sugar, depression screening, and dementia screening. 

Current work in the research group is focussed on implementing design improvements based on the findings of the user trial and as mentioned above, the development of image analysis algorithms for detection and analysis of wound size and chromaticity.

The wound care app, as a prototype mHealth application can contribute to improved documentation and compliance of wound care, while offering distinct benefits in the form of telehealth capabilities and automated data organization and interpretation that provides the user with information easily overlooked in a paper-based file. Results point toward an improved caregiver and patient experience, and concomitant improvements in wound outcomes are anticipated but have not been investigated here. The work is one example of mHealth applications within the broader eHealth spectrum, and one example of telehealth capabilities. 

## Supplementary Files

Supplementary File 1 Supplementary (PDF, 303 KB)
