# Supplementary material for: A mHealth Application for Chronic Wound Care: Findings of a User Trial
Source: Int J Environ Res Public Health. 2013 Nov 19;10(11):6199–214. doi: 10.3390/ijerph10116199 (PMC3863895; doi:10.3390/ijerph10116199)
Supplement: Supplementary File 1 — Supplementary (PDF, 303 KB) [file ijerph-10-06199-s001.pdf]

Marcia Friesen - Riverview Health Centre Survey

This questionnaire asks about your experiences in using the wound care software application on the Smartphone or Tablet device over the past few weeks. Your feedback will be used to further improve and fine-tune the software application's design and functionality. Please note the following:

• Your anonymity is protected. The survey does not ask you for any identifying information, and I request that you do not include any identifying information in your free-form comments. As well, Surveymonkey has been set to not capture your computer's IP address.

• Your confidentiality is protected. As the results are presented to the group during the upcoming focus group to which you are invited, the results will be presented in summary form only. Any presentations or publications arising from this work will likewise only present the results in summary form.

• Your feedback is welcome. A summary of the results will be made available to you via email. To further protect anonymity and confidentiality, you do not need to opt in to receive the results. You wll receive them automatically.

• This survey should take you approximately 20 minutes to complete.

Thank you in advance for your time!

1. The first two questions ask you about your nursing profile.

Years of bedside nursing experience:

- ◯ 1-4 yrs
- ◯ 5-9 yrs
- ◯ 10-14 yrs
- ◯ 15+ yrs

2. Years of experience in personal care home nursing:

- ◯ 1-4 yrs
- ◯ 5-9 yrs
- ◯ 10-14 yrs
- ◯ 15+ yrs

3. Your age:

- ◯ Up to 30 years old
- ◯ 31-40 years old
- ◯ 41-50 years old
- ◯ 51-60 years old
- ◯ over 60 years old

4. Please enter information about your work schedule. In a typical month, how many shifts do you work per week, and how many hours do you work per shift (choose one response for each):

- ☐ In a typical month, I usually work 5 or more shifts per week
- ☐ In a typical month, I usually work 3-4 shifts per week
- ☐ In a typical month, I usually work 1-2 shifts per week
- ☐ My typical shift is 0-4 hours long
- ☐ My typical shift is 5-8 hours long
- ☐ My typical shift is 9-12 hours long

5. How would you rate your own comfort with smartphones & tablets?

- ◯ I'm very tech-savvy.
- ◯ I'm comfortable with common features of phones & tablets.
- ◯ Neutral.
- ◯ Phones & tablets make me a bit nervous.
- ◯ Phones & tablets make me very nervous.

6. This question asks you about the usability of the device (the Smartphone or tablet).

	Very uncomfortable	Somewhat uncomfortable	Neutral	Somewhat comfortable	Very comfortable	N/A
I am comfortable with Smartphone and Tablet interfaces	◯	◯	◯	◯	◯	◯
I am comfortable with touch-screens	◯	◯	◯	◯	◯	◯
Please rate your ability to read easily and enter text easily on the Samsung Smartphone	◯	◯	◯	◯	◯	◯
Please rate your ability to read easily and enter text easily on the Samsung Tablet	◯	◯	◯	◯	◯	◯

Other (please specify)

7. The next three questions ask you about the content of the software application, in terms of how well the software application matches the Braden Scale for risk assessment, the PUSH tool, and the Bates-Jensen tool (paper forms) for charting of wounds and wound care. (Enter N/A if you did not use a particular form).

	Very poorly matched	Poorly matched	Neutral	Well matched	Very well matched	N/A
Braden: How well-matched is the scope and depth of the software application to the Braden Scale tool?	◯	◯	◯	◯	◯	◯
PUSH: How well-matched is the scope and depth of the software application to the PUSH tool?	◯	◯	◯	◯	◯	◯
Bates-Jensen: How well-matched is the scope and depth of the software application to the Bates-Jensen tool?	◯	◯	◯	◯	◯	◯

Other comments

8. Which aspects of the Braden Scale, PUSH tool, and Bates-Jensen tool have been missed or overlooked on the software application? (Enter N/A if you did not use a particular form).

Braden Scale:

PUSH tool:

Bates-Jensen tool:

9. Which aspects of the Braden Scale tool, PUSH tool, and Bates Jensen tool are not clear enough on the software application (i.e. hard to find, less comprehensible, etc.) than on the paper forms, and how could these be made clearer? (Enter N/A if you did not use a particular form).

Braden Scale - aspects that are not clear enough

Braden Scale - ways in which they could be made clearer

PUSH tool - aspects that are not clear enough

PUSH tool - ways in which they could be made clearer

Bates-Jensen tool - aspects that are not clear enough

Bates-Jensen tool - ways in which they could be made clearer

10. Please tell us about your experience with the Treatments section of the software application.

	Strongly disagree	Disagree	Neutral	Agree	Strongly agree	N/A
I always completed the treatments section for each wound assessment	○	○	○	○	○	○
The treatment selections were categorized in a way that made sense to me	○	○	○	○	○	○
In my opinion, the treatment selections were complete, containing all of the typical treatments that one would need to specify when treating a wound	○	○	○	○	○	○
The treatments portion of the assessment form was too overwhelming to use	○	○	○	○	○	○
I would prefer to type in the treatments carried out, rather than picking them from a list of checkboxes	○	○	○	○	○	○

Other comments

11. The rest of the survey asks you or your opinion or impressions of the feature of the software application.

	Difficult to use	Somewhat difficult to use	Neutral	Quite easy to use	Very easy to use	N/A
Password-based login	○	○	○	○	○	○
Entering a new patient record	○	○	○	○	○	○
Finding my existing patient's / resident's wound record	○	○	○	○	○	○
Adding a new wound to the patient's record	○	○	○	○	○	○
Assessing a new wound for the first time	○	○	○	○	○	○
Assessing an existing wound that had been previously assessed	○	○	○	○	○	○

Password-based login — Other comments

Entering a new patient record — Other comments

Finding my existing patient's / resident's wound record — Other comments

Adding a new wound to the patient's record — Other comments

Assessing a new wound for the first time — Other comments

Assessing an existing wound that had been previously assessed — Other comments

12. The software application only presents the screens that are relevant to a particular resident or patient, based on how you respond to each prior screen. Therefore, screen that are not applicable to your resident are not shown to you. We call this 'intuitive guidance' within the software application. Please rate the following:

	Strongly disagree	Disagree	Neutral	Agree	Strongly agree
The screens were presented to me in the order that I expected	○	○	○	○	○
The screens were presented in a logical order	○	○	○	○	○
The screens allowed me to input all the data I expected and needed to input	○	○	○	○	○

Other comments

13. The software application uses Likert-type scales (rating scales, e.g. 1 to 5) wherever possible for consistent data entry. We have minimized the opportunity for free-lance notes, since different healthcare providers may use different wording to describe the same wound conditions.

	Strongly disagree	Disagree	Neutral	Agree	Strongly agree
The Likert-type scales were appropriate in most or all cases.	○	○	○	○	○
The ability to add free-lance notes is important.	○	○	○	○	○
Selecting from a pre-set menu of additional comments would adequately substitute for free-lance notes.	○	○	○	○	○

Other comments

14. The color used to indicate wound severity:

	Strongly disagree	Disagree	Neutral	Agree	Strongly agree
The colors used were appropriate.	○	○	○	○	○
I can easily differentiate between the colors.	○	○	○	○	○

Other comments

15. The software application comes with two built in alerts / notifications. The first one is a notification when a wound assessment is past due, and the second one is a notification when a wound's latest assessment indicates deterioration relative to the previous assessment. These alerts / notifications are shown when you log in to the application. (Please enter N/A if no alerts / notifications were present over the time that you used the software application).

	Strongly disagree	Disagree	Neutral	Agree	Strongly agree	N/A
The alert / notifications were obvious (easy to notice) on the software application.	○	○	○	○	○	○
The alert / notifications were useful	○	○	○	○	○	○
The alert / notifications matched the patient's / resident's actual condition	○	○	○	○	○	○

Other comments

16. This question asks you about the text-based histories of specific wounds, comprised of the Wound History screen and the Week-by-Week comparison feature. (Please enter N/A if you did not use these features).

	Strongly disagree	Disagree	Neutral	Agree	Strongly agree	N/A
History: This presentation is easy to understand.	◯	◯	◯	◯	◯	◯
History: This presentation is helpful in understanding wound progression.	◯	◯	◯	◯	◯	◯
History: This presentation adds to my understanding of the history of the patient's / resident's wounds and wound care, compared to not having this text-based history available.	◯	◯	◯	◯	◯	◯
Week-by-week comparison: This presentation is easy to understand.	◯	◯	◯	◯	◯	◯
Week-by-week comparison: This presentation is helpful in understanding wound progression.	◯	◯	◯	◯	◯	◯
Week-by-week comparison: This presentation adds to my understanding of the history of the patient's / resident's wounds and wound care, compared to not having this text-based history available.	◯	◯	◯	◯	◯	◯

Other comments

17. This question asks you about the graph-based histories of specific wounds on the Wound Graph screens. (Please enter N/A if you did not use these features).

	Strongly disagree	Disagree	Neutral	Agree	Strongly agree	N/A
This presentation is easy to understand.	○	○	○	○	○	○
This presentation is helpful in understanding wound progression.	○	○	○	○	○	○
This presentation adds to my understanding of the history of the patient's / resident's wounds and wound care, compared to not having this graph-based history available.	○	○	○	○	○	○

Other comments

18. This question asks you about taking photos (images) of the wound:

	Never took photos / Photos had a negative impact	Took photos a few times / Photos were not helpful	Took photos quite often / Photos were somewhat helpful	Always took photos / Photos were very helpful	N/A
Tablet users: Did you take photos of the wound?	○	○	○	○	○
Smartphone users: Did you take photos of the wound?	○	○	○	○	○
Tablet users: Were the photos helpful to you in future assessments?	○	○	○	○	○
Smartphone users: Were the photos helpful to you in future assessments?	○	○	○	○	○

If the photos were not helpful, why not?

19. In addition to (or instead of) the current options for a text-based history and a graph-based history, the following display options or modifications would help me to better understand a patient's / resident's wound and wound care status:

20. Please tell us any other impressions or experiences - positive or negative - with the software application that we didn't otherwise cover in this survey. Also, the focus group will be a chance to see a summary of these survey results, and to discuss the experience in person.

Thank you for completing the survey!
